# Selected Emerging Biomarkers in Type 2 Diabetes Mellitus: Clinical Insights and Implications for Precision Care

**DOI:** 10.3390/medicina62010152

**Published:** 2026-01-12

**Authors:** Andra Melissa Entuc, Maria Bogdan, Ianis Kevyn Stefan Boboc, Liliana Mititelu Tartau, Delia Reurean Pintilei, Liliana Lacramioara Pavel, Ana-Maria Pelin, Aurelia Spinei, Liliana Georgeta Foia

**Affiliations:** 1 Grigore T. Popa University of Medicine and Pharmacy Iasi, 700115 Iasi, Romania; melissa.entuc99@gmail.com (A.M.E.); lylytartau@yahoo.com (L.M.T.); georgeta.foia@umfiasi.ro (L.G.F.); 2 Doctoral School of Medicine and Pharmacy, Grigore T. Popa University of Medicine and Pharmacy Iasi, 700115 Iasi, Romania; 3 Department of Pharmacology, Faculty of Pharmacy, University of Medicine and Pharmacy, 200349 Craiova, Romania; bogdanfmaria81@yahoo.com; 4 Faculty of Medicine and Biological Sciences, Ștefan cel Mare University of Suceava, 720229 Suceava, Romania; delia.pintilei@usm.ro; 5 Consultmed Medical Center, 700544 Iasi, Romania; 6 Medical Department, Faculty of Medicine and Pharmacy, Dunarea de Jos University, 800010 Galati, Romania; doctorpavel2012@yahoo.com; 7 Department of Morphological and Functional Sciences, Faculty of Medicine and Pharmacy, “Dunarea de Jos” University, 800010 Galati, Romania; anapelin@gmail.com; 8 Department of Pediatrics, Nicolae Testemitanu State University of Medicine and Pharmacy, MD-2004 Chisinau, Moldova; aurelia.spinei@usmf.md

**Keywords:** novel biomarkers, diabetes mellitus, diagnosis, monitoring, therapy

## Abstract

This paper aims to examine the current landscape of novel biomarkers in diabetes mellitus (DM), with particular emphasis on emerging candidates, and their roles in early diagnosis, monitoring disease progression, risk stratification, and managing complications. Given the global prevalence of DM and its complex pathophysiology, identifying reliable biomarkers is critical for optimizing prevention strategies and personalized treatment approaches. This review highlights the shift from traditional glycemic markers, which remain clinically useful but limited, to a broader array of novel biomarkers that more accurately reflect the complex pathophysiology of DM. In addition to conventional measures, inflammatory and oxidative stress mediators, along with genetic and epigenetic regulators, provide added predictive value for disease susceptibility, progression, and complications. Recent research has identified emerging biomarkers, such as adiponectin, adropin, netrin-1, α-hydroxybutyrate, fetuin-A, lipo-protein(a), and lysophosphatidylcholine, which detect early metabolic imbalances and reveal mechanistic links to insulin resistance, β-cell dysfunction, and vascular injury. Their integration into multimarker panels holds particular promise for precision medicine, supporting tailored prevention, targeted therapy, and improved outcomes for individuals with prediabetes and DM.

## 1. Introduction

**Figure 1:**
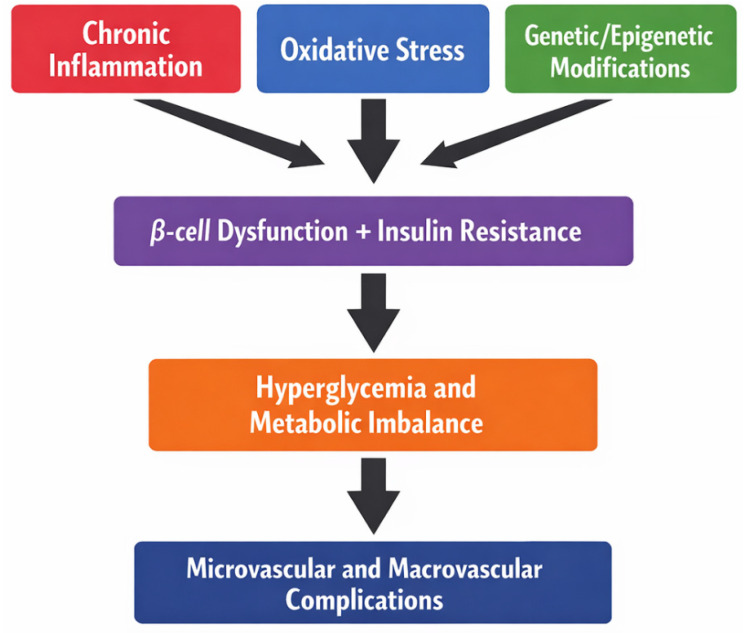
Mechanistic schema of DM progression.

Diabetes mellitus (DM) is a global public health challenge, currently affecting millions and projected to reach 853 million adults by 2050 [1]. This chronic metabolic disorder, characterized by persistent hyperglycemia from impaired insulin secretion, insulin resistance, or both, impacts multiple organ systems and significantly reduces quality of life [2,3,4]. DM is heterogeneous: type 1 (T1DM) is autoimmune and usually arises in childhood, causing absolute insulin deficiency [5], while type 2 (T2DM), accounting for over 90% of cases, involves genetic and environmental factors, insulin resistance, and impaired insulin secretion, and is increasingly seen in younger populations due to obesity and sedentary lifestyles [2,6]. Gestational DM also elevates long-term T2DM risk for mother and child [7] (Figure 1).

Key risk factors for T2DM include unhealthy diets, physical inactivity, obesity, tobacco and alcohol use, chronic stress, urbanization, aging, and sedentary lifestyles [8,9]. Over 240 million individuals remain undiagnosed, delaying treatment and increasing complication risk [10]. If poorly controlled, DM leads to severe microvascular complications (retinopathy, nephropathy, neuropathy) and macrovascular complications (myocardial infarction, stroke, peripheral artery disease), imposing substantial physical and psychosocial burdens [11,12,13].

**Figure 2:**
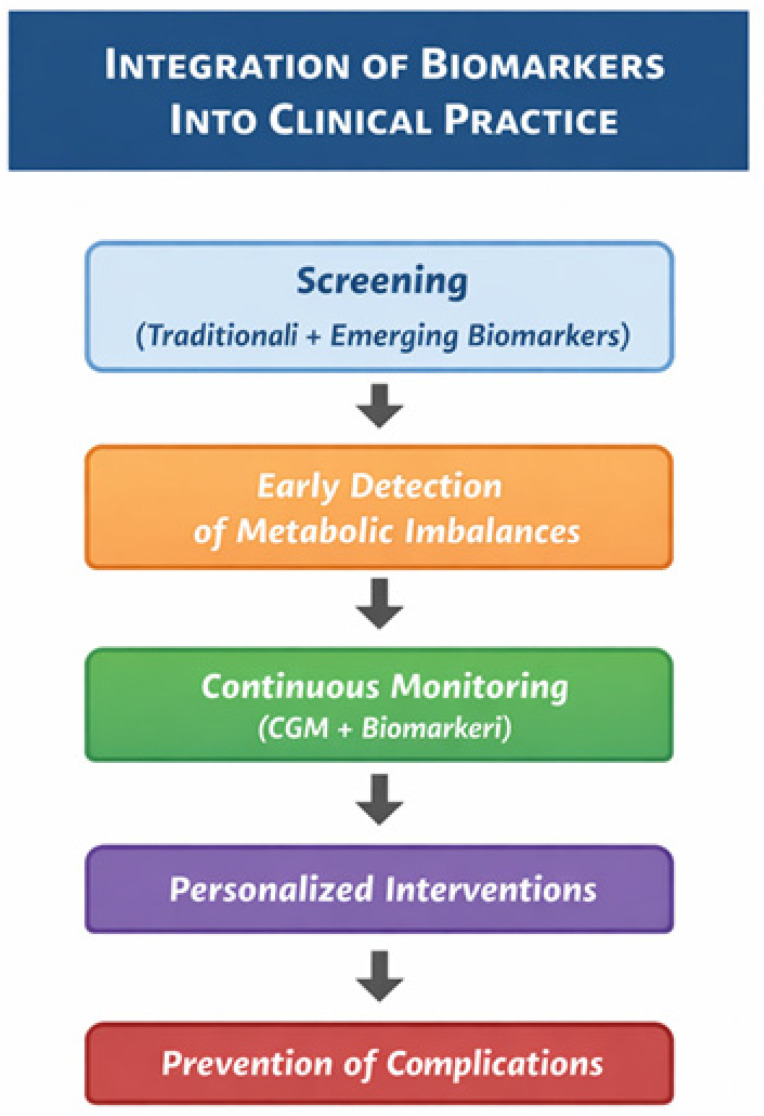
Clinical implementation of biomarkers in DM care. CGM—continuous glucose monitoring.

Health systems, particularly in low- and middle-income countries, are increasingly overwhelmed by the growing need for DM care, prevention programs, and patient education [14] (Figure 2).

Against this backdrop, early identification of prediabetes is vital to halting the progression to overt DM. A range of biomarkers is currently used for this purpose, including biochemical indicators (e.g., fasting glucose, HbA1c), as well as inflammatory, genetic, and epigenetic markers. Recent research highlights the growing value of these tools in assessing individual susceptibility to DM. For example, certain genetic polymorphisms and epigenetic modifications may indicate a heightened risk for insulin resistance long before clinical symptoms become apparent [15,16].

The development of prediabetes is multifactorial and complex, influenced by a combination of genetic predisposition, impaired insulin secretion, insulin resistance, glucotoxicity, lipotoxicity, chronic low-grade inflammation, oxidative stress, and a decline in both the mass and function of pancreatic β-cells [17,18]. Preventing the progression from prediabetes to T2DM is a key public health priority. Early interventions, such as dietary improvements, increased physical activity, weight loss, metabolic monitoring, patient education, and accessible community healthcare, significantly reduce diabetes risk and improve health outcomes [19]. Prevention strategies are implemented at three levels: (1) primary prevention promotes healthy lifestyles in the general population [20]; (2) secondary prevention identifies high-risk individuals using screening tools like fasting glucose, HbA1c, and oral glucose tolerance tests (OGTT) [21]; (3) tertiary prevention manages diagnosed patients to prevent complications through monitoring, tailored therapy, education, and multidisciplinary care, as exemplified by the U.S. National DPP, which reduced T2DM risk by up to 58% in high-risk individuals [22].

The biomarkers included in this review were selectively chosen based on several predefined criteria: (i) mechanistic relevance to core pathophysiological processes involved in DM, such as insulin resistance, inflammation, oxidative stress, hepatic dysfunction, and early organ injury; (ii) availability of human clinical data linking these biomarkers to glycemic status, cardiometabolic risk, or diabetic complications; (iii) potential applicability within precision medicine frameworks, including risk stratification, phenotyping, and treatment monitoring; (iv) emerging but incomplete evidence, which highlights both opportunities and current limitations for clinical translation. This focused approach was intended to provide depth and clinical relevance rather than an exhaustive catalog of all proposed diabetes biomarkers.

This article is a narrative review aimed at synthesizing current evidence on classical and emerging biomarkers in DM, with a particular focus on their clinical relevance in T2DM and related cardiometabolic conditions. The literature was identified through systematic searches of major biomedical databases, including PubMed/MEDLINE, Scopus, and Web of Science, covering publications from January 2008 to August 2025. Additional relevant articles were identified by manual screening of reference lists from key reviews and original studies.

The search strategy combined terms related to DM and biomarkers, including: *“diabetes mellitus”, “type 2 diabetes”, “prediabetes”, “biomarkers”, “novel biomarkers”, “adiponectin”, “adropin”, “α-hydroxybutyrate”, “fetuin-A”, “lipoprotein(a)”, “netrin-1”, “lysophosphatidylcholine”, “GPLD1”, “clinical studies”, “risk prediction”, and “diabetic complications”*.

Studies were selected based on their relevance to the clinical application of biomarkers in DM, including diagnosis, risk stratification, disease monitoring, and prediction of complications. Priority was given to human clinical studies, observational and interventional trials, systematic reviews, and meta-analyses published in peer-reviewed journals. Experimental animal studies were included only when they provided important mechanistic insights supporting clinical findings. Articles not available in English, case reports with limited clinical relevance, and studies lacking sufficient methodological detail were excluded.

## 2. Novel Biomarkers in DM

Early diagnosis and monitoring of DM and prediabetes are crucial to preventing complications that impair quality of life, with biomarkers providing key insights into metabolic state, disease progression, and therapeutic response [23,24,25]. HbA1c remains the gold standard for assessing chronic glycemic control [26,27], but its diagnostic accuracy improves when combined with alternative biomarkers such as fructosamine, glycated albumin (GA), and 1,5-anhydroglucitol (1,5-AG), a multimarker strategy particularly useful for early detection of prediabetes and individualized T2DM prevention [28,29,30].

Continuous glucose monitoring (CGM) has emerged as a valuable tool for real-time assessment of glycemic variability and metabolic control in both T1DM and T2DM. By providing continuous measurements of interstitial glucose levels, CGM allows for early detection of hyperglycemia and hypoglycemia, assessment of glucose trends, and evaluation of therapeutic interventions. The integration of CGM data with traditional biomarkers and clinical parameters enhances precision monitoring, supports individualized treatment adjustments, and may improve long-term outcomes in patients with DM.

Beyond these classic biomarkers, DM is also associated with chronic inflammation and oxidative stress, reflected by markers such as C-reactive protein (CRP), fibrinogen, interleukin-1 beta (IL-1β), interleukin-6 (IL-6), interleukin-18 (IL-18), interleukin-1 receptor antagonist (IL-1RA), plasminogen activator inhibitor-1 (PAI-1), superoxide dismutase (SOD), malondialdehyde (MDA), glutathione peroxidase, glutathione, 8-hydroxy-2′-deoxyguanosine (8-OHdG), and the accumulation of advanced glycation end-products (AGEs), all of which contribute to disease progression and complications [31,32,33,34].

Moreover, genetic variants such as single nucleotide polymorphisms (SNPs) in transcription factor 7-like 2 (TCF7L2), solute carrier family 30 member 8 (SLC30A8), potassium voltage-gated channel subfamily J member 11 (KCNJ11), fat mass and obesity-associated protein (FTO), peroxisome proliferator-activated receptor gamma (PPARG), and cyclin-dependent kinase 5 regulatory subunit associated protein 1-like 1 (CDKAL1), together with insertions/deletions and copy number variations, modulate diabetes susceptibility and treatment response [35,36,37,38,39,40], while epigenetic mechanisms including DNA methylation, histone modifications, and microRNAs regulating genes like Pdx1, IGF2, INS, and PPARGC1A further contribute to disease risk, progression, and personalized therapy [41,42,43,44,45,46] (Table 1).

In addition to established markers, several novel biomarkers have recently been identified, offering greater sensitivity and specificity for detecting prediabetes, monitoring DM progression, and predicting complications [47]. Monitoring metabolic, inflammatory, and oxidative stress biomarkers is critical for preventing both acute and chronic complications [18,48,49]. Advances in laboratory technology now allow precise measurement of these markers, enhancing clinical decision-making. Integrating traditional and emerging biomarkers improves early diagnosis, supports personalized treatment, and strengthens risk assessment, ultimately leading to better patient outcomes [23].

### 2.1. Adiponectin

Adiponectin is an adipocyte-derived hormone with a central role in glucose and lipid metabolism, insulin sensitivity, and inflammation. Unlike most adipokines, circulating adiponectin levels decrease with increasing adiposity and insulin resistance [50,51]. Through activation of AMP-activated protein kinase (AMPK) and peroxisome proliferator-activated receptor-α (PPAR-α) pathways, adiponectin enhances fatty acid oxidation, suppresses hepatic gluconeogenesis, and promotes glucose uptake in peripheral tissues [52]. In addition to its metabolic actions, adiponectin exerts anti-inflammatory and anti-atherogenic effects by inhibiting proinflammatory cytokines and protecting endothelial function [53,54,55]. These biological properties link reduced adiponectin levels to the pathophysiology of obesity, insulin resistance, and T2DM, as well as to the development of vascular complications [55,56,57].

Despite extensive supporting evidence, the clinical application of adiponectin remains limited by significant inter-assay variability, population-specific reference ranges, and the lack of universally accepted cut-off values. These methodological and biological differences complicate result interpretation and hinder its routine use in clinical practice. Although adiponectin reflects key metabolic and inflammatory pathways, its incremental predictive value beyond established cardiometabolic risk factors has been inconsistent across studies, limiting its standalone prognostic utility.

### 2.2. Adropin

Adropin is a secreted peptide involved in energy homeostasis, endothelial function, and immune regulation [58,59,60]. It participates in metabolic signaling pathways that influence glucose utilization, lipid metabolism, and vascular integrity [61,62]. Experimental data suggest that adropin contributes to endothelial protection, modulation of inflammatory responses, and maintenance of tissue homeostasis. Given that DM is characterized by chronic hyperglycemia, oxidative stress, and low-grade inflammation, alterations in adropin signaling may reflect early metabolic and vascular dysfunction. These mechanistic properties position adropin as a potential link between metabolic imbalance, inflammation, and organ damage in DM [63,64,65].

Nevertheless, further large-scale, longitudinal studies are required to validate these findings and to clarify adropin’s clinical utility as a reliable biomarker and potential therapeutic target in DM.

### 2.3. α-Hydroxybutyrate

α-Hydroxybutyrate (α-HB) is an emerging metabolic biomarker produced during threonine and methionine catabolism and involved in hepatic glutathione synthesis [66]. Variations in its concentration may reflect complex metabolic imbalances, such as oxidative stress and amino acid metabolism disturbances, which are typical in the early stages of DM [67]. Its levels rise under oxidative stress and redox imbalance, processes common in early dysglycemia [18,68,69]. Clinically, α-HB is a sensitive early marker of insulin resistance and prediabetes, often preceding changes in fasting glucose or HbA1c. Elevated α-HB is linked to impaired glucose tolerance, reduced insulin sensitivity, and higher risk of progression to T2DM [70,71,72]. Longitudinal studies confirm its predictive value for future dysglycemia, even in normoglycemic individuals. Its utility lies in enabling early risk stratification, capturing dynamic oxidative stress and metabolic disruption missed by traditional markers, and providing a non-invasive diagnostic option through mass spectrometry-based blood tests [67,68,73]. However, widespread adoption is limited by cost, lack of assay standardization, and confounding effects of other oxidative stress-related conditions [48,67]. Current research is exploring α-HB within multi-marker panels alongside amino acids, lipids, and acylcarnitines to improve DM risk assessment, and efforts are ongoing to adapt it for point-of-care and personalized prevention strategies [74].

Despite its promise as an early marker of insulin resistance, the clinical application of α-hydroxybutyrate (α-HB) remains limited by several factors. Measurement currently relies on mass spectrometry-based platforms, which are costly, not universally available, and lack standardized analytical protocols across laboratories. Moreover, no universally accepted cut-off values have been established for defining abnormal α-HB levels in prediabetes or diabetes. Reported associations may also be confounded by conditions characterized by oxidative stress or altered amino acid metabolism, including liver disease, malignancy, and inflammatory disorders. Importantly, although α-HB often rises before changes in fasting glucose or HbA1c, its incremental predictive value beyond established clinical variables has not been consistently demonstrated in large, diverse populations. Therefore, current evidence supports α-HB as a promising but still investigational biomarker.

### 2.4. Fetuin-A

Fetuin-A is a hepatokine predominantly synthesized by the liver, with important roles in insulin signaling, inflammation, and mineral metabolism [75,76,77,78]. It interferes with insulin receptor autophosphorylation, thereby contributing to insulin resistance, and is closely associated with hepatic steatosis and metabolic dysfunction [79,80,81]. Fetuin-A also participates in inflammatory pathways and vascular calcification, indicating a complex, context-dependent role in cardiometabolic disease. Through these mechanisms, altered Fetuin-A levels reflect early metabolic disturbances and may contribute to the progression of T2DM and its complications [82,83,84,85,86,87].

The translation of Fetuin-A into clinical practice is constrained by similar challenges, including assay variability, ethnic and population-dependent distributions, and the absence of standardized threshold values. These factors reduce comparability across studies and restrict its broader clinical implementation. While Fetuin-A offers complementary insights into insulin resistance, inflammation, and vascular calcification, evidence regarding its added predictive value beyond conventional risk markers remains variable, highlighting the need for further validation.

### 2.5. Glycosylphosphatidylinositol-Specific Phospholipase D1 (GPLD1)

Glycosylphosphatidylinositol-specific phospholipase D1 (GPLD1) is an enzyme that releases GPI-anchored proteins from cell membranes, influencing signaling, immunity, and metabolic regulation [88,89]. Elevated serum GPLD1 is associated with insulin resistance, systemic inflammation, β-cell dysfunction, lipid abnormalities, adipose dysfunction, and metabolic dysfunction-associated steatotic liver disease (MASLD, formerly NAFLD), all key features of T2DM and metabolic syndrome [90,91,92,93]. As a serum-based marker, GPLD1 offers promise for early detection of metabolic dysregulation, often rising before overt hyperglycemia. It reflects chronic low-grade inflammation and β-cell stress, supporting its role in identifying individuals at high risk of T2DM and prediabetes [94]. GPLD1 levels may also be useful for monitoring responses to anti-inflammatory or insulin-sensitizing therapies.

Clinically, GPLD1 can be measured through ELISA or proteomics, enabling non-invasive application [89,95]. Given its involvement in glucose metabolism, lipid signaling, and immune regulation, GPLD1 represents a novel biomarker for early diagnosis, risk stratification, and personalized management of DM and its complications.

Although GPLD1 has emerged as a potential biomarker of metabolic inflammation and insulin resistance, its clinical utility is not yet well established. Available studies are relatively few, often cross-sectional, and involve heterogeneous populations. Assay standardization and reference ranges for circulating GPLD1 are not clearly defined, and levels may be influenced by comorbid conditions such as non-alcoholic fatty liver disease, obesity, and chronic inflammatory states. Furthermore, it remains unclear whether GPLD1 provides meaningful incremental information beyond conventional markers of glycemic control and cardiometabolic risk. Larger longitudinal studies are needed to clarify its diagnostic and prognostic relevance in diabetes.

### 2.6. Lipoprotein(a)

Lipoprotein(a) [Lp(a)] is a plasma lipoprotein consisting of an LDL particle bound to apolipoprotein(a), giving it proatherogenic and proinflammatory properties [96,97,98]. Levels are largely genetically determined, show little variation over time, and are minimally influenced by lifestyle or glycemic control [99,100]. In T2DM, Lp(a) is gaining importance as an independent cardiovascular risk factor, since traditional markers like LDL-C and HbA1c do not fully explain residual ASCVD risk [101,102]. Mechanistically, Lp(a) promotes atherogenesis via impaired fibrinolysis, endothelial dysfunction, and vascular inflammation [103]. Elevated concentrations are often seen in diabetic patients with coronary artery disease, although its relationship with glycemia is inconsistent, reflecting its distinct metabolic profile [104,105,106,107].

Unlike other cardiometabolic biomarkers, Lp(a) is not significantly reduced by lifestyle or standard antidiabetic therapy. Instead, pharmacological strategies directly targeting Lp(a) show greater promise [108]. Some evidence also suggests sex-based differences, with higher levels observed in women with DM, though clinical significance remains unclear [109,110]. Overall, Lp(a) is a stable, genetically driven biomarker valuable for cardiovascular risk assessment in DM, especially in patients with good metabolic control but persistent ASCVD risk. Measuring Lp(a) can guide the use of targeted therapies and improve precision in cardiovascular prevention [111,112].

However, further prospective studies are needed to define clinically relevant thresholds, clarify sex-specific implications, and determine how Lp(a) measurement can be optimally integrated into risk stratification and treatment algorithms for patients with DM.

### 2.7. Lysophosphatidylcholine (L-GPC)

Lysophosphatidylcholine (L-GPC) is a bioactive phospholipid produced by hepatic phospholipase A2 and lecithin-cholesterol acyltransferase, involved in lipid metabolism, signaling, and membrane integrity, while also influencing insulin-mimetic pathways [113,114]. Altered L-GPC levels are associated with insulin resistance, β-cell dysfunction, and early metabolic imbalance, positioning it as a potential biomarker for prediabetes detection and monitoring [115,116]. L-GPC profiles provide insight into lipid dysregulation and subclinical inflammation that precede overt hyperglycemia, aiding early risk stratification [117]. Its fluctuations also reflect immune and endothelial dysfunction relevant to DM progression.

Clinically, L-GPC can be measured using lipidomics platforms like mass spectrometry, offering a sensitive, reproducible, and minimally invasive diagnostic tool [118,119]. Shifts in L-GPC levels may also indicate therapeutic response, supporting personalized treatment and prevention strategies [118].

The clinical interpretation of L-GPC is challenged by substantial heterogeneity in lipidomic methodologies, species-specific measurements, and analytical platforms. Differences in sample handling, mass spectrometry techniques, and data normalization complicate cross-study comparisons. In addition, circulating L-GPC levels are influenced by diet, inflammation, lipid disorders, and cardiovascular disease, raising concerns about specificity for diabetes-related metabolic dysfunction. While altered L-GPC profiles have been associated with insulin resistance and early dysglycemia, evidence regarding their added predictive value over traditional metabolic markers remains limited. Consequently, L-GPC currently represents an exploratory biomarker that requires further standardization and validation before routine clinical use.

### 2.8. Netrin-1

Netrin-1 is a laminin-related guidance protein with immunomodulatory, anti-inflammatory, and tissue-protective properties [120,121,122,123,124]. Beyond its role in neuronal development, netrin-1 regulates immune cell migration, endothelial integrity, and inflammatory signaling. In the context of DM, dysregulation of netrin-1 pathways may contribute to chronic inflammation, vascular dysfunction, and neural injury. These biological actions suggest a mechanistic link between netrin-1 signaling and the development of microvascular and macrovascular complications in DM [125,126,127,128,129,130,131,132].

Interpretation of netrin-1 as a diabetes biomarker is complicated by its context-dependent regulation and dual biological roles. Circulating and urinary netrin-1 levels may reflect compensatory, protective responses in early disease stages, while declining levels are observed in advanced complications, leading to apparently conflicting findings across studies. Differences in sample type (serum vs. urine), disease stage, and comorbidities further contribute to heterogeneity. In addition, standardized assays and clinically validated cut-off values are lacking. Although netrin-1 shows promise for early detection of diabetic complications, particularly nephropathy, its incremental predictive value over established renal and vascular markers remains insufficiently defined.

## 3. Clinical Studies on the Use of Novel Biomarkers in the Management of DM

The clinical studies summarized in Tables S1–S5 were selected to illustrate representative and clinically meaningful evidence for each biomarker, rather than to provide an exhaustive or quantitative synthesis. Given the heterogeneity of study designs, populations, and outcome measures, no formal meta-analysis was performed.

Most available clinical evidence derives from cohorts with T2DM.

**Figure 3:**
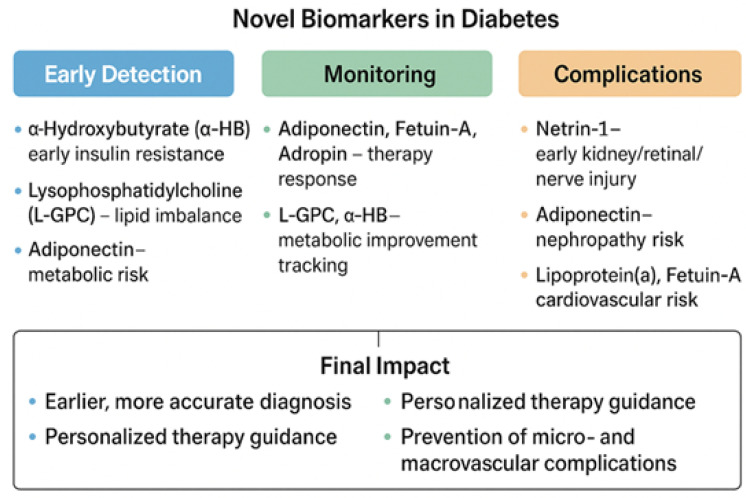
Clinical applications of novel biomarkers in DM.

Emerging diabetes biomarkers carry substantial clinical implications, as they have the potential to enhance early disease detection, improve risk stratification, reveal underlying pathophysiological heterogeneity, and guide more precise, individualized therapeutic interventions (Figure 3).

### 3.1. Adiponectin

Clinical studies consistently demonstrate lower circulating adiponectin levels in individuals with prediabetes and T2DM compared with normoglycemic controls. Reduced adiponectin has been associated with poorer glycemic control, increased insulin resistance, and higher cardiometabolic risk. In patients with diabetic complications, altered adiponectin levels have been linked to nephropathy, cardiovascular disease, and overall disease severity. Interventional studies indicate that pharmacological treatments and lifestyle modifications may increase adiponectin concentrations, often paralleling improvements in metabolic parameters. Collectively, these findings support adiponectin as a clinically relevant biomarker for risk stratification, disease progression, and therapeutic monitoring in DM [133,134,135,136,137,138,139,140,141,142,143].

### 3.2. Adropin

Clinical evidence indicates that circulating adropin levels are altered across different stages of DM and its complications. Lower serum adropin concentrations have been reported in individuals with T2DM, particularly in those with diabetic nephropathy, cardiovascular disease, or metabolic-associated fatty liver disease. Several studies demonstrate correlations between adropin levels and markers of renal function, vascular injury, and systemic inflammation. Moreover, pharmacological interventions, including incretin-based therapies and SGLT2 inhibitors, have been shown to modulate adropin levels in parallel with metabolic improvement. These findings suggest that adropin may serve as a clinically informative biomarker for disease severity, complication risk, and therapeutic response in DM [144,145,146,147,148,149,150,151,152,153,154,155].

### 3.3. Fetuin-A

Clinical studies consistently associate elevated circulating Fetuin-A levels with insulin resistance, prediabetes, and T2DM. Increased concentrations have been observed prior to overt hyperglycemia, supporting its role as an early marker of metabolic risk. Fetuin-A levels have also been linked to the presence and severity of diabetic complications, including vascular disease and diabetic foot syndrome. Interventional studies indicate that lifestyle changes, pharmacological therapies, and bariatric surgery can reduce Fetuin-A levels, often preceding or exceeding improvements in conventional metabolic markers. These findings highlight Fetuin-A as a responsive biomarker for metabolic dysfunction, complication risk assessment, and monitoring of therapeutic efficacy [156,157,158,159,160,161,162].

### 3.4. Lipoprotein(a)

Lipoprotein(a) [Lp(a)] is an independent cardiovascular risk factor in T2DM, largely resistant to lifestyle or glycemic interventions, but responsive to certain pharmacologic therapies [163,164,165,166,167,168,169,170,171]. Clinical studies demonstrate that rosiglitazone, ERN/LRPT (extended-release niacin/laropiprant), and antihypertensive combination therapy significantly reduce Lp(a) levels, alongside improvements in oxidative stress, lipid profiles, and glycemic control [163,164,165]. PCSK9 inhibitors, such as alirocumab and evolocumab, also markedly lower Lp(a) by 19–38% without negatively impacting glycemic parameters, highlighting their cardiovascular benefit in diabetic patients [166,168].

Dietary interventions, including almond supplementation, produced non-significant reductions in Lp(a), while exercise interventions, including aerobic and blood flow-restricted resistance training, failed to modify Lp(a) despite improvements in other cardiometabolic parameters [167,170]. Cross-sectional studies indicate slightly elevated Lp(a) in T2DM compared to non-diabetic individuals, with higher levels observed in those with coexisting coronary artery disease, supporting Lp(a)’s role as a predictor of cardiovascular complications independent of traditional metabolic markers [169,170]. Overall, Lp(a) is a stable, genetically determined biomarker in T2DM, useful for cardiovascular risk stratification, with targeted pharmacologic interventions being the most effective strategy for reducing its levels [163,164,165,166,167,168,169,170,171].

### 3.5. Netrin-1

Clinical studies indicate that circulating and urinary netrin-1 levels are altered in DM and its complications. Reduced serum netrin-1 has been reported in patients with cardiovascular disease, diabetic neuropathy, and retinopathy, whereas increased urinary netrin-1 may precede albuminuria in diabetic nephropathy, suggesting early renal injury. Changes in netrin-1 levels have also been observed in response to therapeutic interventions, supporting its potential utility in monitoring disease progression and treatment response. Overall, netrin-1 emerges as a promising biomarker for early detection and risk stratification of diabetic complications across multiple organ systems [172,173,174,175,176,177,178].

## 4. Precision Care Implications of Novel Biomarkers in DM

From a precision medicine perspective, novel diabetes biomarkers offer the opportunity to move beyond a one-size-fits-all approach based solely on glycemic thresholds. Rather than replacing established markers such as HbA1c, these biomarkers may complement traditional measures by capturing distinct pathophysiological domains, including insulin resistance, inflammation, oxidative stress, hepatic dysfunction, and early organ injury. In clinical practice, such biomarkers could be integrated into multimarker risk scores to improve early detection of prediabetes, identify high-risk individuals with normoglycemia, and stratify patients according to their predominant metabolic or complication-related sub-phenotypes. Moreover, dynamic changes in biomarkers such as adiponectin, adropin, or Fetuin-A may support individualized treatment monitoring and guide the intensity of pharmacological or lifestyle interventions. While most applications remain investigational, this integrative, multimodal approach aligns with the goals of precision diabetes care by enabling earlier intervention, targeted prevention strategies, and more personalized management pathways (Table 2).

## 5. Limitations

This review has several limitations that should be acknowledged. First, the heterogeneity of the included studies, regarding population characteristics, study design, biomarker measurement techniques, and outcome definitions, limits the ability to draw uniform conclusions about the clinical utility of certain biomarkers. Additionally, many novel biomarkers discussed remain in the early stages of validation, with limited longitudinal and large-scale population data to support their routine clinical use.

Second, although clinical studies were referenced to illustrate the practical application of several biomarkers, the review does not include a quantitative synthesis (e.g., meta-analysis) to assess effect sizes or comparative performance. This restricts the strength of evidence regarding biomarker accuracy, sensitivity, and predictive value.

Despite their potential, several challenges must be addressed before novel biomarkers can be widely adopted in clinical practice. These include the need for standardized measurement protocols, validation across diverse populations, cost-effectiveness assessments, and clear regulatory pathways. Moreover, healthcare providers require appropriate infrastructure, digital tools, and clinical training to interpret and apply biomarker data effectively. The expanding field of biomarker research holds transformative potential for diabetes care. By enabling earlier diagnosis, more accurate prognostication, and individualized treatment, biomarkers offer a path toward more efficient and effective clinical management. Achieving this vision will require sustained interdisciplinary collaboration among researchers, clinicians, technologists, and policymakers.

## 6. Conclusions

This literature review highlights the growing importance of both classical and emerging biomarkers in the diagnosis, monitoring, and personalized management of DM, particularly in T2DM and related cardiometabolic conditions. As the global prevalence of DM continues to rise, early detection and targeted intervention are critical to reducing disease burden and improving patient outcomes.

Traditional biomarkers such as HbA1c, fructosamine, and glycated albumin remain foundational tools in assessing glycemic control, yet they are not without limitations, particularly in complex or comorbid clinical scenarios. In response, a new generation of biomarkers has emerged, including adiponectin, netrin-1, and α-hydroxybutyrate, offering improved sensitivity and pathophysiological specificity. These novel markers provide insights into insulin resistance, β-cell dysfunction, inflammation, oxidative stress, and early organ damage. Incorporating these biomarkers into clinical practice has the potential to enhance diagnostic precision, enable risk stratification, and guide personalized therapeutic strategies. Furthermore, several biomarkers may also serve as therapeutic targets, opening new avenues for drug development and preventive interventions.

Clinical studies support the utility of biomarker-based approaches across various stages of diabetes care, from identifying individuals with prediabetes to monitoring the effectiveness of pharmacologic and lifestyle interventions. However, challenges remain in terms of standardization, validation, accessibility, and cost-effectiveness.

Looking forward, the integration of multimarker panels with digital health tools and machine learning algorithms could greatly advance precision medicine in diabetes. For such approaches to be successfully implemented, interdisciplinary collaboration and supportive health policy frameworks are essential.

Biomarker-driven strategies hold significant promises to transform the management of DM, enabling earlier diagnosis, more effective treatment, and improved long-term outcomes.

## Figures and Tables

**Table 1:** Major biomarkers for DM diagnosis and risk assessment.

Biomarker Category	Clinical Utility	Examples
**Glycemic/Classical**	Diagnosis; long-term glycemic monitoring	Fasting plasma glucose; 2 h OGTT; HbA1c; Fructosamine; Glycated albumin (GA); 1,5-Anhydroglucitol
**Inflammatory**	Cardiometabolic risk assessment; complication prediction	CRP; IL-6; IL-1β; IL-18; IL-1RA; TNF-α; PAI-1
**Oxidative Stress**	Complications detection; endothelial dysfunction	Malondialdehyde (MDA); Glutathione peroxidase (GPx); Superoxide dismutase (SOD); Total glutathione
**Genetic & Epigenetic**	Prediction; susceptibility; pharmacogenomics	SNPs (TCF7L2, SLC30A8, CDKAL1, PPARG, FTO); DNA methylation (Pdx1, IGF2, INS, PPARGC1A); Histone modifications; Circulating miRNAs (miR-375, miR-34a, miR-126)
**Novel/Emerging**	Early detection; prediction of insulin resistance; organ-specific complications	Metabolic: adiponectin; α-hydroxybutyrate (α-HB); lysophosphatidylcholine (L-GPC) Endothelial/neural: adropin; netrin-1 Hepatokine: fetuin-A Lipoprotein-associated: lipoprotein(a) Proteomic: GPLD1

**Table 2:** Potential precision care applications of novel biomarkers in DM.

Biomarker	Key Pathophysiological Domain	Potential Precision Care Use-Cases
Adiponectin	Insulin sensitivity, inflammation	Early identification of insulin-resistant phenotype; cardiometabolic risk stratification; monitoring response to lifestyle and insulin-sensitizing therapies
Adropin	Endothelial function, energy homeostasis	Identification of vascular/metabolic dysfunction phenotypes; monitoring cardiometabolic and renal complication risk; treatment response marker
α-Hydroxybutyrate	Oxidative stress, early dysmetabolism	Early detection of insulin resistance and prediabetes; inclusion in multimarker metabolic risk panels
Fetuin-A	Hepatic insulin resistance, inflammation	Stratification of liver-related metabolic phenotypes; prediction of diabetes progression and vascular complications; monitoring response to metabolic interventions
GPLD1	Inflammation, β-cell stress	Experimental marker for early metabolic dysfunction; potential component of proteomic risk signatures
Lipoprotein(a)	Atherothrombotic risk	Identification of residual cardiovascular risk in diabetes; guidance for intensified cardiovascular prevention
Lysophosphatidylcholine (L-GPC)	Lipid metabolism, insulin resistance	Exploratory marker for early metabolic imbalance; integration into lipidomic risk models
Netrin-1	Inflammation, tissue injury	Early detection of diabetic complications (renal, neural, vascular); potential monitoring marker for organ-specific damage

## Data Availability

No new data were created or analyzed in this study.

## Supplementary Materials

The following supporting information can be downloaded at: https://www.mdpi.com/article/10.3390/medicina62010152/s1, Table S1: Clinical studies on the use of adiponectin in the management of DM; Table S2: Clinical studies on the use of adropin in the management of DM; Table S3: Clinical studies on the use of Fetuin-A in the management of DM; Table S4: Clinical studies on the use of lipoprotein(a) in the management of DM; Table S5: Clinical studies on the use of Netrin-1 in the management of DM.
