# Supplementary material for: Selected Emerging Biomarkers in Type 2 Diabetes Mellitus: Clinical Insights and Implications for Precision Care
Source: Medicina (Kaunas). 2026 Jan 12;62(1):152. doi: 10.3390/medicina62010152 (PMC12843815; doi:10.3390/medicina62010152)
Supplement: Supplementary file 1 [file medicina-62-00152-s001.zip › medicina-4046313-supplementary.pdf]

Table S1. Clinical studies on the use of adiponectin in the management of DM.

First author / Year	Pathology	Patient group (Total / Male/ Female)	Patient age (years) mean ± SD	Adiponectin	Marker values (µg/mL)	Timing of analysis	Medication	Other patologies
Abdella / 2018 [133]	T2DM	575 FDR (undiagnosed risk)<br>376 known T2DM patients	FDR: Normal: 28.2±8.6<br>Undiagnosed DM: 32±9.7<br>T2DM:<br><53 mmol/mol: 55.4±13.5<br>>53 mmol/mol: 57.8±9.4	Plasma	FDR:<br>Normal: 8.57±4.42<br>Undiagnosed DM: 6.92±2.86<br>T2DM:<br><53 mmol/mol: 8.58±4.5<br>>53 mmol/mol: 7.37±4.35	Baseline	None at time of study	-
Alnaggar / 2019 [134]	T2DM ± Diabetic nephropathy	44 patients<br>(26 M, 18 F)	35-55	Serum	Normoalbuminuric: 61.22±44.18 (9.5–170);<br>Microalbuminuric: 140.74±93.37 (34–358)	Baseline	Not detailed (all had T2DM treatment)	Hypertension<br>Dyslipidaemia
Hartman / 2020 [135]	T2DM<br>MASLD	316 patients<br>Placebo: 51 (29 M, 22 F)<br>TIR 1mg: 52 (29 M, 23 F)<br>TIR 5mg: 55 (34 M, 21 F)<br>TIR 10mg: 51 (30 M, 21 F)<br>TIR 15 mg: 53 (22 M, 31 F)<br>DUL 1.5 mg: 54 (24 M, 30 F)	Placebo:56.6±8.9<br>TIR 1mg: 57.4±8.9<br>TIR 5mg: 57.9±8.2TIR 10mg: 56.5±9.9<br>TIR 15 mg: 56.0±7.6<br>DUL 1.5 mg: 58.7±7.8	Serum	Baseline:<br>Placebo: 4.0±0.4<br>TIR 1mg: 5.4±0.4<br>TIR 5mg: 4.2±0.4<br>TIR 10mg: 4.3±0.4<br>TIR 15 mg: 5.1±0.5<br>DUL 1.5 mg: 4.5±0.41<br>Change from Baseline at 26 Weeks:<br>Placebo: -0.1±0.2<br>TIR 1mg: -0.1±0.2<br>TIR 5mg: 0.4±0.2<br>TIR 10mg: 0.9±0.2<br>TIR 15 mg: 0.9±0.3<br>DUL 1.5 mg: 0.3±0.2	Baseline and after 26 months	TIR (1, 5, 10, 15 mg), DUL (1.5 mg), or placebo; metformin allowed if stable dose;	
Bidulescu / 2020 [136]	T2DM	3363 patients (1230 M, 2133 F)<br>T2DM: 584 (216 M, 368 F)<br>Control: 2779 (1014 M, 1765 F)	T2DM:<br>54.7±0.5<br>Control: 52.5±0.2	Plasma	T2DM: 4.092 ± 0.115 (3.203±0.149 M, 4.625±0.155 F)<br>Control: 5.566±0.077 (4.061±0.095 M, 6.439±0.103 F)	Baseline	1404 Antihypertensive, 6 Antihyperlipidemic<br>275 Antihypertriglyceridemi c	Hypertension and Dyslipidemia
Werida / 2021 [137]	T2DM<br>Dyslipidemia	160 patients<br>ATOR 80 (38 M, 42 F)<br>ROSU 80 (36 M, 44 F)	ATOR 54.38±5.84<br>ROSU 55.45±5.94	Serum	Baseline<br>ATOR: 1.36 ±0.46<br>ROSU: 1.49 ±0.53<br><br>At 6 month:<br>ATOR: 2.40 ±0.74	Baseline and after 6 months	Baseline used medications<br>ATOR:<br>38 Glimepiride<br>28 Glibenclamide<br>14 Vildagliptin	Hypertension<br>OB

					ROSU: 2.95 ±0.94		8 ACEIs<br>7 ARBs<br><br>ROSU:<br>37 Glimepiride<br>24 Glibenclamide<br>19 Vildagliptin<br>11 ACEIs<br>10 ARBs	
Pena / 2023 [138]	T2D risk in adolescents + OB	64 patients (38 M, 26 F)<br><br>INT: 40 (24 M, 16 F)<br>UC: 24 (15 M, 9 F)	13.3±1.4<br><br>INT: 13.4±1.4<br>UC 13.2±1.4	Serum	Baseline:<br>1.5 ±1.7<br>INT: 1.67±0.12<br>UC 1.28±0.11<br>At 6 months:<br>INT: 1.42±0.11<br>UC 1.21±0.11	Baseline and after 6 months	No DM medications; only lifestyle or usual care interventions	-
Reddy / 2023 [139]	T2DM + MASLD	42 patients (17 M, 25 F)<br>LFD: 22 baseline<br>19 at the end<br>MedDiet:<br>19 baseline<br>18 at the end	52.3 ± 12.6	Serum	Baseline:<br>11.3 ± 13.9<br>LFD: 17.3 ± 14.4<br>MedDiet: 13.7 ± 9.2<br>At 12 weeks:<br>LFD: 19.5 ± 21.0<br>MedDiet: 17.0 ± 12.6	Baseline and after 12 weeks	35 patients on prescription medication; 24 patients took supplements; stable during intervention	-
Hayashishita / 2024 [140]	T2DM	24 patients T2DM 17 (14 M, 3 F); Controls 7 (4 M, 3F)	T2DM: 62.2±23.0<br>Controls: 39.3±13.3	Serum	T2DM: 5.9 ± 4.0<br>Controls: 10.9 ± 10.1;	Blood sample collected prior to insulin administration	T2DM:<br>8 Antihypertensives, 8 Hyperlipidemic agent<br>Antidiabetic Medication Use:<br>6 Metformin<br>5 Insulin<br>2 SGLT2i<br>5 DPP-4i<br>2 Sulfonylureas	Hypertension and Dyslipidemia
Yaikwawong / 2024 [141]	T2DM + OB	229 patients<br>Curcumin:<br>114 (62 M, 73 F)<br>Placebo:<br>115 (54 M, 80 F)	≥35 years	Serum	Curcumin group:<br>0: 8.75 (7.04–10.56)<br>3: 8.91 (7.04–10.56)<br>6: 13.73 (5.32–52.05)<br>9: 13.74 (5.46–53.02)<br>12: 14.51 (5.78–54.67)<br>Placebo group:<br>0: 8.85 (7.04–10.56)<br>3: 8.89 (7.04–10.56)<br>6: 10.13 (3.81–29.28)<br>9: 10.38 (3.43–27.45)	Baseline, 3, 6, 9, 12 months	Metformin (monotherapy); no change allowed antihypertensive and antidyslipidemic medications<br>Curcumin	Hypertension and Dyslipidemia

					12: 10.36 (3.21–26.21)			
Lee / 2025<br>[142]	T2DM +<br>MASLD	44 patients<br><br>PIO: 14 (8 M, 6 F)<br>EMPA: 16 (9 M, 7 F)<br>PIO+EMPA: 14 (7 M, 7 F)	53.8 ± 12.5<br><br>PIO: 49.6±10.2<br>EMPA: 56.3±10.1<br>PIO+EMPA: 55.1±16.4	Serum	Baseline:<br>2.6 [2.1, 3.9]<br>PIO: 2.5 [2.2, 3.6]<br>EMPA: 2.6 [2.1, 3.5]<br>PIO+EMPA: 3.6 [1.8, 4.1]<br>At 24 weeks:<br>PIO: 10.1 [17, 13.7]<br>EMPA: 7.0 [5.8, 11.4]<br>PIO+EMPA: 14.8 [9.8, 18.6]	Baseline and after 24 weeks	PIO 15 mg/day, EMPA 10 mg/day, or both in combination<br>Antidiabetic Medication Use: Metformin<br>Sulfonylurea<br>DPP-4I<br>Insulin	-
Barb / 2025<br>[143]	T2DM +<br>MASLD	38 patients (21 M, 17 F)	Placebo: 58 ± 11; LAN: 61 ± 7	Serum	Baseline:<br>Placebo: 5.0 ± 3.7<br>LAN: 4.5 ± 2.8<br>Fold change from baseline in Adiponectin:<br>Placebo: 1.0 [0.6; 1.4]<br>LAN: 2.4 [2.0; 2.7]	Baseline and after 24 weeks	LAN 800 mg/day or placebo;<br>Antidiabetic Medication Use: Metformin, GLP-1Ras, SGLT2i,<br>DPP-4i, Sulfonylureas	

ACS – Acute Coronary Syndrome, ATOR – Atorvastatine, DM – Diabetes Mellitus, DPP-4i – dipeptidyl peptidase-4 inhibitors, DUL – Dulaglutide, EMPA – empagliflozin, F – Female, FDR – First-degree relatives with a T2DM diagnosed patient, GLP-1RAs – glucagon-like peptide-1 receptor agonists, HbA1c – glycated hemoglobin, INT – Intervention Group, LAN – Lanifibranor, LVESD – Left Ventricular End-Systolic Diameter, M – Male, MASLD – Metabolic Dysfunction-Associated Steatotic Liver Disease, OB – Obese, PIO – pioglitazone, ROSU – Rosuvastatine, SGLT2i – sodium–glucose cotransporter-2 inhibitors, T2DM – Type 2 Diabetes Mellitus, TIR – Tirzepatide, UC – Usual Care Group.

Table S2. Clinical studies on the use of adropin in the management of DM.

First author / Year	Pathology	Patient group (Total / Female / Male)	Patient age (years) mean ± SD	Adropin	Marker values (ng/mL)	Timing of analysis	Medication	Other patologies
Hu / 2016<br>[144]	T2DM	81 Control (46 M, 35 F)<br><br>110 NOA (58 M, 52 F)<br>95 MIA (49 M, 46 F)<br>40 MAA (21 M, 19 F)	Control 58.35±7.80<br><br>NOA 57.52±11.33<br>MIA 58.45±13.16<br>MAA 59.92±10.78	Serum	Control 3.71 (2.82–4.56)<br><br>NOA 3.17 (2.63–3.68)<br>MIA 2.85 (2.32–3.27)<br>MAA 2.56 (2.07–2.85)	Baseline	Antidiabetic drugs	-
Es-haghi / 2021<br>[145]	T2DM<br>DKD	44 T2DM with DKD (21 M, 23 F)<br>45 T2DM (23 M, 22 F)<br>45 Control (22 M, 23 F)	T2DM with DKD 51.43±6.04<br>T2DM 47.60±4.87<br>Control 48.67±5.08	Serum	T2DM with DKD 2.77±0.36<br>T2DM 3.35±0.36<br>Control 4.21±0.52	Baseline	-	-
Li / 2021<br>[146]	MASLD and its progression to T2DM	62 patients<br>30 MASLD (10 M, 20 F)<br>MASLD-NGT (5 M, 8 F)<br>MASLD-T2DM (5 M, 12 F)	MASLD: 35.1±9.5<br>MASLD-NGT: 32.2±9.5<br>MASLD-T2DM: 37.3±9.1<br>Control: 35.1±4.8	Serum	MASLD: 2.02±2.92<br>Control: 5.52±0.65<br><br>MASLD-NGT: 4.00±3.52<br>MASLD-T2DM: 0.51±0.73	Baseline	No drug intervention was given in patients with T2DM.	-

		32 control (11 M, 11 F)						
Smith / 2022 [147]	Vascular insulin resistance due to short-term obesogenic lifestyle	36 patients (18 M, 18 F)	M: 25±1<br>F: 22±1	Plasma	M: ↓ from baseline (exact ng/mL not specified)<br>F: unchanged	Before and after 10-day intervention	None reported; healthy volunteers	-
Wang / 2022 [148]	T2DM	35 T2DM: (21 M, 14 F)<br>28 Control (16 M, 12 F)	T2DM: 50.31±13.43<br>Control: 50.25±13.49	Serum	Baseline:<br>T2DM: 3.12±0.73<br>Control: 5.90±1.22<br><br>At 17 weeks:<br>T2DM: 4.97±1.01	Baseline and after 17 weeks	Sitagliptin 100 mg/day	-
Wei / 2022 [149]	Carotid atherosclerosis in T2DM	503 patients (297 M, 206 F)<br>223 WCP (133 M, 90 F)<br>280 CP (164 M, 116 F)	WCP: 51.6±9.8<br><br>CP: 61.5±10.1	Serum	WCP: 20.5±4.0<br>CP: 20.6±3.7	Baseline	Insulin 36 WCP, 58 CP<br>OADs 123 WCP, 173 CP<br>Statins WCP 6, CP 17	Hypertension WCP 75, CP 131<br>MASLD 116 WCP, 117 CP
Aydın / 2022 [150]	COVID-19<br>T2DM	100 patients (40 M, 60 F)	T2DM: 23–70<br>COVID: 23–87<br>COVID+DM: 38–92<br>Control: 31–75	Serum	Control: 0.6426±0.387<br><br>T2DM: 0.2561±0.07085<br><br>COVID: 0.3673±0.1797<br><br>COVID+T2DM: 0.1624±0.1151	Baseline	OADs or insulin	Hyperlipidemia<br>Depression<br>Parkinson's disease<br>Dry eye syndrome
Berezin / 2023 [151]	T2DM<br>CHF	417 patients<br>(231 M, 186 F)	53 (41–64)<br>M: 52 (40–63)<br>F: 54 (42–66)	Serum	T2DM: 2.37 (1.91–2.75)<br>M: 2.11 (1.82–2.68)<br>F: 2.69 (2.32–3.04)<br><br>At 6 months:<br>T2DM: 3.00 (2.68–3.36)<br>M (male): 2.60 (2.07–3.21)<br>F (female): 3.65 (3.40–3.89)	Baseline and at 6 months	Dapagliflozin 10 mg/day<br><br>Other medication:<br>198 ACEi, 106 M, 92 F<br>67 ARBs, 34 M, 33 F<br>165 ARNi, 92 M, 73 F<br>372 beta-blockers, 201 M, 171 F<br>59 ivabradine, 32 M, 27 F<br>75 calcium channel blocker, 38 M, 37 F<br>283 MRA, 150 M, 133 F<br>358 loop diuretic, 196 M, 162 F<br>367 antiplatelet, 201 M, 166 F<br>51 anticoagulant, 34 M, 17 F	346 dyslipidemia, 198 M, 150 F<br>352 hypertension, 192 M, 160 F<br>141 stable CAD, 75 M, 66 F<br>135 MIA, 79 M, 56 F<br><br>334 LV hypertrophy, 191 M, 143 F<br>112 DKD 1–3 grades, 62 M, 50 F<br>57 atrial fibrillation, 34 M, 23 F

							387 metformin, 210 M, 177 F<br>408 statins, 226 M, 182 F	
Zhang / 2023 [152]	T2DM with MASLD	22 T2DM (17 M, 5 F)<br><br>22 Controls (17 M, 5 F)	38.55±9.33<br><br><br>40.18±10.64	Serum	Baseline<br>T2DM: 2.79±0.47<br>Control: 3.27±0.79<br><br>At 12 weeks:<br>T2DM: 3.65 (3.20, 3.85)	Baseline and after 12 weeks	Liraglutide (0.6 to 1.8 mg/day SC over 12 weeks)	-
Berezina / 2023 [153]	T2DM, CHF	480 patients (272 M, 208 F)<br><br>88 PCE (51 M, 37 F)<br><br>392 PWCE (221M, 171F)	53 (40–67)<br><br>PCE<br>56 (46–68)<br><br>PWCE<br>51 (40–64)	Serum	2.37 (1.90–2.75)<br><br>PCE<br>2.07 (1.80–2.35)<br><br>PWCE<br>2.69 (2.10–3.17)<br><br>At 52 weeks:<br><br>PCE<br>1.98 (1.61–2.27)<br>PWCE<br>2.85 (2.34–3.11)	Baseline and 52 weeks	296 ACEi, 52 PCE, 244 PWCE<br>72 ARBs, 16 PCE, 56 PWCE<br>112 ARNi, 22 PCE, 90 PWCE<br>427 beta-blockers, 77 PCE, 350 PWCE<br>93 ivabradine, 12 PCE, 81 PWCE<br>131 calcium channel blockers, 27 PCE, 104 PWCE<br>147 MRA, 24 PCE, 123 PWCE<br>383 loop diuretics, 75 PCE, 308 PWCE<br>166 antiplatelet, 33 PCE, 133 PWCE<br>105 anticoagulants, 41 PCE, 64 PWCE<br>480 metformin, 88 PCE, 392 PWCE<br>429 SGLT2i, 76 PCE, 353 PWCE<br>453 statins, 78 PCE, 375 PWCE	385 dyslipidemia, 70 PCE, 315 PWCE<br>307 hypertension, 55 PCE, 252 PWCE<br>166 stable coronary artery disease, 38 PCE, 128 PWCE<br>19 dilated cardiomyopathy, 5 PCE, 14 PWCE<br>105 atrial fibrillation, 37 PCE, 68 PWCE<br>56 paroxysmal/persistent atrial fibrillation, 18 PCE, 38 PWCE<br>49 permanent atrial fibrillation, 19 PCE, 30 PWCE<br>215 abdominal obesity, 40 PCE, 175 PWCE<br>382 left ventricular hypertrophy, 72 PCE, 310 PWCE<br>118 DKD grades 1–3, 31 PCE, 87 PWCE
Berezina / 2024 [154]	Newly diagnosed prediabetes Post STEMI	498 patients (263 M, 235 F)<br><br>126 COG (77 M, 49 F)<br>372 COFR (186 M, 186 F)	63 (51–75)<br><br>COG 65 (52–77)<br>COFR<br>62 (50–75)	Serum	Baseline:<br>2.96 (1.92–4.30)<br><br>COG: 2.14 (1.80–2.83)<br><br>COFG: 3.64 (2.70–5.58)	At baseline	316 ACEi, 80 COG, 236 COFR<br>78 ARBs, 21 COG, 57 COFR<br>456 beta-blockers, 115 COG, 341 COFR	425 dyslipidemia, 113 COG, 312 COFR<br>353 hypertension, 88 COG, 265 COFR<br>216 chronic HF, 55 COG, 161 COFR

							82 ivabradine, 21 COG, 61 COFR<br>61 calcium channel blockers, 16 COG, 45 COFR<br>43 thiazide-like diuretics, 11 COG, 32 COFR<br>216 loop diuretics, 55 COG, 161 COFR<br>173 MRA, 39 COG, 134 COFR<br>429 antiplatelet agents, 112 COG, 317 COFR<br>90 anticoagulants, 24 COG, 66 COFR<br>116 metformin, 28 COG, 88 COFR<br>95 GLP-1RAs, 18 COG, 77 COFR<br>297 SGLT2i, 59 COG, 238 COFR<br>498 statins, 126 COG, 372 COFR	43 HFpEF, 11 COG, 32 COFR<br>173 HFmrEF, 44 COG, 129 COFR<br>90 atrial fibrillation, 24 COG, 66 COFR<br>211 smoking, 57 COG, 154 COFR<br>194 abdominal obesity, 53 COG, 141 COFR<br>467 left ventricular hypertrophy, 118 COG, 349 COFR<br>131 DKD grades 1–3, 37 COG, 94 COFR
Chen / 2025 [155]	T2DM<br>DKD	58 T2DM:<br>40 ADKD (27 M, 13 F)<br>18 EDKD (11 M, 7 F)<br><br>9 Control (4 M, 5 F)	T2DM:<br>ADKD 67.10±10.13<br>EDKD 52.89±12.95<br>Control 56.44±11.18	Serum	T2DM:<br>ADKD 6.848±1.287<br>EDKD 5.380±1.826<br>Control 3.470±1.284<br><br>DKD progression: 7.52±0.84<br>Non- DKD<br>Progression: 6.15±1.66	Baseline	T2DM:<br><br>metformin, 17 ADKD, 17 EDKD<br>sulfonylurea, 27 ADKD, 9 EDKD<br>TZD, 9 ADKD, 5 EDKD<br>DPP4i, 20 ADKD, 5 EDKD<br>GLP-1RAs, 8 ADKD, 4 EDKD<br>SGLT2i, 7 ADKD, 8 EDKD<br>insulin injection, 21 ADKD, 6 EDKD<br>use of anti-hypertensive agent, 36 ADKD, 10 EDKD<br>statin, 27 ADKD, 9 EDKD<br>Control:	

							use of anti-hypertensive agent 3	

ACEi – Angiotensin-Converting Enzyme Inhibitor, ADKD – Advanced Diabetic Kidney Disease, ARBs – Angiotensin II Receptor Blocker, ARNi – Angiotensin Receptor–Neprilysin Inhibitor, CAD – Coronary Artery Disease, CHF – Chronic Heart Failure, DPP-4i – dipeptidyl peptidase-4 inhibitors, DKD – Diabetic Kidney Disease, COFG – Clinical Outcomes Free Group, COG – Clinical Outcomes Group, CP – With Carotid Plaque, EDKD – Early Diabetic Kidney Disease, F – Female, GLP-1RAs – glucagon-like peptide-1 receptor agonists, HF – Heart Failure, IGT – Impaired Glucose Tolerance, LV – Left Ventricle, MAA – Macroalbuminuria, MASLD – Metabolic Dysfunction-Associated Steatotic Liver Disease, M – Male, MIA – Microalbuminuria, MRA – Mineralocorticoid Receptor Antagonist, NGT – Normal Glucose Tolerance, NOA – Normoalbuminuria, OADs – Oral Antidiabetic Drugs, PCE – Patients with Composite Endpoint, PWCE – Patients without Composite Endpoint, SC – subcutaneous, SGLT2i – sodium–glucose cotransporter-2 inhibitors, STEMI – ST Elevation of Myocardial Infarction, TZD – thiazolidinediones, T2DM – Type 1 Diabetes Mellitus, WCP – Without Carotid Plaque.

Table S3. Clinical studies on the use of Fetuin-A in the management of DM.

First author / Year	Pathology	Patient group (Total / Male/ Female)	Patient age (years) mean ± SD	Fetuin-A	Marker values (µg/mL)	Timing of analysis	Medication	Other patologies
Wedick / 2011<br>[156]	T2DM	45 patients<br>(16 M, 29 F)	CafC 38.7 (7.3)<br>DCafC 41.9 (14.6)<br>NCaf 41.2 (17.4)	Serum	Baseline:<br>CafC 246.1 (45.3)<br>DCafC 270.7 (37.4)<br>NCaf 280.2 (56.9)<br><br>At 8 weeks:<br>CafC 261.52 (228.83, 298.84)<br>DCafC 233.71 (201.54, 270.99)<br>NCaf 291.07 (212.94, 333.94)	Baseline and Week 8	None allowed	Overweight only
Özenç / 2013<br>[157]	T2DM	137 patients: (M: 107 / F: 30)<br><br>Control (23 M, 8 F)<br>T2DM (37 M, 12 F)<br>T2DMWDF (47 M, 10 F)	Control: 51 (36–78)<br>T2DM: 59 (39–87)<br>T2DMWDF: 64 (42–82)	Serum	Control: 0.036<br>T2DM: 0.085<br>T2DMWDF: 0.123	Baseline	T2DM: Oral antidiabetic and insulin treatment<br>T2DMWDF Insulin	Atherosclerosis in peripheral arteries (graded by ultrasound);
Jüllig / 2014<br>[158]	T2DM	15 patients<br>8 GBP (0 M, 8 F)<br>7 SG (1 M, 6 F)	GBP: 41.0 ± 3.1;<br>SG: 46.8 ± 2.9	Serum	Decreased to 75% of baseline after GBP	3 days before and 3 days after surgery	metformin monotherapy pre-surgery, stopped post-surgery	OB
Otten / 2018<br>[159]	T2DM	26 patients<br><br>13 PD (9 M, 4 F)<br>13 PD-EX (8 M, 5 F)	PD 60 (54–64)<br>PD-EX 61 (58–67)	Plasma	Decreased by 11% in PD group; unchanged in PD-EX group.	Before and after 12-week intervention	Diet ± metformin (patients were treated with diet and/or metformin only)	OB
Krajnc / 2019<br>[160]	Coronary calcification in T2DM	45 patients (23 M, 22 F)	59 ± 8 (M: 60 ± 7, F: 58 ± 8)	Serum	Baseline: 0.0262 ± 0.0055<br><br>At 18 months: 0.0280 ± 0.0070	Baseline and after 18 months	35 Metformin<br>20 Insulin<br>11 Insulin + metformin<br>37 Statin 34 ACEI/sartan	OB
Khalili / 2019<br>[161]	T2DM	40 patients (20 M, 20 F)<br>Placebo (7 M, 13 F)<br>Probiotic (7 M, 13 F)	Placebo 45.00 (5.37)<br>Probiotic 43.95 (8.14)	Serum	Baseline:<br><br>Placebo: 139.95 (45.36)	Baseline and at 8 weeks	15 Glibenclamide (7 placebo, 8 probiotic)	-

		10 M / 10 F per group)			Probiotic :119.53 (46.95)<br><br>At 8 weeks:<br>Placebo: 145.00 (45.86)<br>Probiotic: 107.63 (41.79)		40 Metformin	
Nada / 2025 [162]	T2DM	60 patients<br>Placebo (8 M, 12 F)<br>Curcumin (9 M, 11 F)<br>Fenofibrate (6 M, 14 F)	Placebo (56.15 ± 5.02)<br>Curcumin 57.40 ± 6.48<br>Fenofibrate 56.30 ± 8.16	Serum	Baseline:<br>Placebo 132.78 ± 38.61<br>Curcumin 140.56 ± 43.15<br>Fenofibrate 157.03 ± 66.49<br><br>At 3 weeks:<br>Placebo 141.15 (116.08-207.49)<br>Curcumin 121.2 (111.35-147.5)<br>Fenofibrate 94.40 (74.55-128.60)	Baseline and at 3 months	Glimepirid	Hypertension

TCafC – Caffeinated Coffee, DCafC – Decaffeinated Coffee, GBP – Gastric Bypass, NCaf – No Coffee, OB – Obesity, PD – Paleolithic Diet Group, PD-EX – Paleolithic Diet and Exercise Group, SG – Sleeve Gastrectomy, T2DM – Type 2 Diabetes Mellitus, T2DMWDF – Type 2 Diabetes Mellitus with Diabetic Foot.

Table S4. Clinical studies on the use of lipoprotein(a) in the management of DM.

First author / Year	Pathology	Patient group (Total / Male/ Female)	Patient age (years) mean ± SD	Lipoprotein A (Lp (a))	Marker Values (mg/dL)	Timing of analysis	Medication	Other patologies
Atamer / 2013 [163]	T2DM	50 patients (30 M, 20 F)	58.7 ± 9.2	Serum	Baseline: 28.20±6.47<br><br>At 3 months: 19.32±3.74	Before and at 3 months	Metformin, sulfonylurea, insulin + rosiglitazone	-
Bays / 2015 [164]	T2DM	796 patients<br>ERN/LRPT (≤6.8%): (134 M, 83 F)<br>Placebo (≤6.8%): (115 M, 60 F)<br>ERN/LRPT (>6.8%): (122 M, 93 F)<br>Placebo (>6.8%): (99 M, 62 F)	ERN/LRPT (≤6.8%): 62.59±9.15<br>Placebo (≤6.8%): 61.61±9.41<br>ERN/LRPT (>6.8%): 61.47±9.29<br>Placebo (>6.8%): 62.32 ± 9.37	-	Baseline:<br>ERN/LRPT (≤6.8%): 10.00<br>Placebo (≤6.8%): 11.00<br>ERN/LRPT (>6.8%): 8.50<br>Placebo (>6.8%): 10.00<br><br>At 12 weeks, treatment with ERN/LRPT significantly reduced Lp (a) by approximately 22–26%, regardless of baseline HbA1c or FPG levels.<br><br>At 36 weeks, no quantitative data on Lp (a) are provided, so the	Baseline and at 12, 36 weeks	Fish oils Statins<br>Fibrates (gemfibrozil, fenofibrate)<br>Ezetimibe<br>Ezetimibe / simvastatin combination tablet<br>Bile acid sequestrants<br><br>Antidiabetic medication	Dyslipidemia

					progression beyond week 12 cannot be evaluated.			
Derosa / 2016 [165]	T2DM	221 patients Olmesartan: 74 (36 M, 38 F) Amlodipine: 72 (35 M, 37 F) Olmesartan / Amlodipine: 75 (35 M, 40 F)	Adults ≥18	Plasma	Baseline:<br>Olmesartan: 45.2±39.5<br>Amlodipine: 43.4±38.7<br>Olmesartan / Amlodipine: 45.1±39.7<br><br>At 12 months:<br>Olmesartan: 39.4±36.1<br>Amlodipine: 42.5±38.1<br>Olmesartan / Amlodipine: 32.5±31.8	Baseline and at 12 months	Olmesartan<br><br>Amlodipină<br><br>Olmesartan+Amlodipină	Hypertension
Leiter / 2017 [166]	T2DM<br>T1DM	441 T2DM:<br>ARLI (161 M, 133 F)<br>Placebo (78 M, 69 F)<br>76 T1DM:<br>ARLI (29 M, 22 F)<br>Placebo (17 M, 8 F)	<65<br>T2DM:<br>ARLI 143<br>Placebo 73<br>T1DM:<br>ARLI 42<br>Placebo 19<br>≥65 to <75<br>T2DM:<br>ARLI 126<br>Placebo 55<br>T1DM:<br>ARLI 8<br>Placebo 6<br>≥75<br>T2DM:<br>ARLI 25<br>Placebo 19<br>T1DM:<br>ARLI 1<br>Placebo 0	Plasma	Baseline:<br>T2DM:<br>ARLI 16.0 (5.0:55.0)<br>Placebo 14.0 (5.0:38.0)<br>T1DM:<br>ARLI 17.0 (6.0:28.0)<br>Placebo 12.0 (4.0:37.0)<br><br>Change from Baseline to Week 24<br>T2DM:<br>ARLI −19.0% ± 1.6%<br>Placebo −0.5% ± 2.2%<br>T1DM:<br>ARLI −23.0% ± 3.8%<br>Placebo −4.3% ± 5.3%	Baseline and at 24 weeks	Insulin (all), Statins (majority), Ezetimibe metformin<br>Other antihyperglycaemic drugs alirocumab	High CV risk
Gulati / 2017 [167]	T2DM	50 patients (27 M, 23 F)	45.8±9.3	Serum	Baseline:<br>21.8 (9.38–122)<br>At 24 weeks:<br>19.8 (9.38–111)	Baseline and at 24 weeks	Metformin	Cardiovascular Risk
Lorenzatti / 2018 [168]	T2DM	981 patients<br>657 EVO (290 M, 367 F)<br>324 Placebo (199 M, 195 F)	EVO 62 (33-80)<br>Placebo 62 (35-80)	Serum	Baseline:<br>EVO 69.4 (94.3) nmol/L<br>Placebo 69.4 (93.6) nmol/L<br><br>At 12 weeks:<br>EVO Q2W: −35.9%	Baseline, and at 12 weeks	Alpha-glucosidase inhibitors<br>– Placebo: 36<br>– EVO: 110<br>Biguanides<br>– Placebo: 223	Hypertension<br>Cerebrovascular or peripheral arterial disease<br>CAD

					EVO QM: −37.9%<br>Placebo: +26.5%		– EVO: 454<br>DPP-4i<br>– Placebo: 32<br>– EVO: 56<br>GLP-1RAs<br>– Placebo: 5<br>– EVO: 7<br>Insulin<br>– Placebo: 91<br>– EVO: 211<br>Meglitinides<br>– Placebo: 12<br>– EVO: 31<br>SGLT2i<br>– Placebo: 10<br>– EVO: 19<br>Sulfonylureas<br>– Placebo: 120<br>– EVO: 223<br>TZD<br>– Placebo: 14<br>– EVO: 19<br>Other<br>– Placebo: 3<br>– EVO: 11	
Mahmoodi / 2022<br>[169]	T2DM	253 patients<br>125 T2DM (70 M. 55, F)<br>128 control (67 M , 61 F)	47.79±5.65	Serum	Baseline<br>Total Participants:<br>T2DM: 68.04±3.74 ng/mL<br>Control: 66.98±2.57 ng/mL<br>M:<br>T2DM: 64.45±4.40 ng/mL<br>Control: 68.18±3.79 ng/mL<br>F:<br>T2DM: 72.61±6.37 ng/mL<br>Control: 65.65±3.42 ng/mL	Baseline	Other antihyperglycaemic drugs	Cardiometabolic risk
Qiu / 2024<br>[170]	T2DM	896 patients<br>T2DM+CAD: 217 (140 M, 77 F)<br>Controls: 679 (431 M, 248 F)	T2DM+CAD: 57.61 ± 10.13<br>Control: 55.81 ± 9.17	Plasma	T2DM+CAD:<br>16.53±20.19<br>Control: 14.00±15.08	Baseline	DM medication	CAD

Ma / 2024 [171]	T2DM	93 patients 31 Control (11 M, 20 F)<br>30 AE (16 M, 14 F)<br>32 BFR-RT (16 M, 16 F)	Control 56.29±5.92<br>AE 57.73±5.85<br>BFR-RT 57.56±4.85	Serum	Baseline:<br>Control 111.58±45.32<br>AE 115.60±46.06<br>BFR-RT 110.03±43.22<br>At 3 months:<br>Control 112.95±45.23<br>AE 116.64±44.02<br>BFR-RT 105.44±40.69<br>At 6 months:<br>Control 114.09±45.71<br>AE 108.93±44.77<br>BFR-RT 101.34±43.61	Baseline and at 3 and 6 months	Glucose-lowering medication<br>Medications used for dyslipidemia<br>Medications used for blood pressure	ASCVD<br>Dyslipidemia<br>Hypertension

AE – aerobic exercise, ARLI – arlilocumab, ASCVD – atherosclerotic cardiovascular disease,, BFR-RT – blood flow-restrictive resistance training, CAD – coronary artery disease, DM – Diabetes Mellitus, DPP-4i – dipeptidyl peptidase-4 inhibitors, ERN/LRPT – extended-release niacin/laropiprant, EVO – evolocumab, FPG – fasting plasma glucose, GLP-1RAs – glucagon-like peptide-1 receptor, HbA1c – glycated hemoglobin, Lp(a) – lipoprotein A, Q2W – every 2 weeks, QM – every month, SGLT2i – sodium–glucose cotransporter-2 inhibitors, TZD – thiazolidinediones, T1DM – type 1 diabetes mellitus, T2DM – type 2 diabetes mellitus, Lp(a) concentrations are reported in nmol/L, ng/mL or mg/dL, as provided in the original studies; direct conversion between units is not recommended due to apo(a) isoform variability.

Table S5. Clinical studies on the use of Netrin-1 in the management of DM.

First author / Year	Pathology	Patient group (Total / Female / Male)	Patient age (years) mean ± SD	Netrin-1	Marker values (pg/mL)	Timing of analysis	Medication	Other patologies
Okutucu / 2021 [172]	DR (NPDR and PDR) in T2DM	50 patients with T2DM (10 NPDR, 13 PDR) + 27 controls	NPDR 66.70 ± 12.37 PDR 67.85 ± 8.93 Control 65.74 ± 7.17	Serum	Baseline:<br>NPDR: 253 ± 75<br>PDR: 318 ± 68<br>Control: 983 ± 664<br>1 week:<br>NPDR: 416.3 ± 123.0<br>PDR: 476.6 ± 225.0<br>4 weeks:<br>NPDR: 747.5 ± 89.6<br>PDR: 676.6 ± 187.5	At the time of ophthalmic evaluation, 1 and 4 weeks after BCZ injection	BCZ injection	Hypertension
Inderjeet / 2022 [173]	T2DM ± ACS	84 patients: T2DM with ACS: 42 (31 M, 11 F)<br>T2DM 42 (29 M, 13F)	T2DM with ACS: 58.29 ± 10.24<br>T2DM 56.02 ± 7.22	Serum<br>Netrin-4 (serum)	Netrin-1:<br>T2DM with ACS 510.47 ± 85.72<br>T2DM 623.25 ± 68.55<br>Netrin-4:<br>T2DM with ACS 43.96 ± 9.80<br>T2DM 142.71 ± 24.01	Within 24–30 h of T2DM with ACS (patienst); routine OPD visit (T2DM)	-	-
Garcia Galindo / 2022 [174]	OB and newly diagnosed T2DM	90 patients<br>Healthy: 30 (1 1F / 9 M)	Healthy: 22.8 ± 4.3<br>OB: 21.9 ± 4.5	Serum	Healthy: 130 ± 60<br>OB: 150 ± 70<br>T2DM: 330 ± 220	Single time-point blood sample	None reported	

		OB: 30 (14 F / 6 M)<br>T2DM: 30 (10 F / 10 M)	T2DM: 51.3 ± 5.5					
Mondal / 2024 [175]	T2DM, DN (with and without small fiber loss)	117 patients:<br>45 controls 42 DN- 30 DN+	Control: 43.1 ± 10.5; DN-: 49.4 ± 10.3; DN+: 53.2 ± 8.0	Serum	Control: 1,034 ± 352<br>DN-: 810.2 ± 308.6<br>DN+: 522.3 ± 182	Single time-point blood sample	-	-
Mentxaka / 2024 [176]	T2DM ± OB	91 patients<br>LN: 18 (10 F/8 M)<br>OB-NG: 32 (25 F/7 M)<br>OB-T2DM: 41 (28 F/13 M)	LN: 42 ± 5<br>OB-NG: 40 ± 3<br>OB-T2DM: 47 ± 2	Serum	LN: 578 ± 33<br>OB-NG: 924 ± 64<br>OB-T2DM: 841 ± 50	Single time-point blood sample	None reported explicitly (patients with T2DM were untreated)	-
Matter / 2025 [177]	T1DM with DKA and transient renal tubulopathy	40 patients (36 F, 4 M)	10.59 ± 2.17	Urine	Day 1: Median 723.08 (range: 210.5–4223)<br>Day 3: Median 764.33 (range 518.73–1159)<br>Day 14: Median 180.3 (range: 153.48–255.43)	Day 1 (on DKA presentation)<br>Day 3<br>Day 14 (follow-up)	Insulin	-
Chaitra / 2025 [178]	T2DM ± DFS	260 patients: 130 DF<br>130 T2DM	-	Serum	Median Netrin-1:<br>DF = 180.83 (IQR 118.93–280.45)<br>T2DM = 164.37 (IQR 108.73–210.72)	Single time-point blood sample	-	-

ACS – Acute coronary syndrome, BCZ – bevacizumab, DKA – Diabetic ketoacidosis, DN – Diabetic Neuropathy, DR – Diabetic Retinopathy, DFS – Diabetic Foot Syndrome, F – Female, IQR – interquartile range, LN – lean, M – Male, NPDR – non-proliferative diabetic retinopathy, OB – obese, OB-NG – obese with normoglycaemia, OB-T2DM – obese with Type 2 Diabetes Mellitus, PDR – proliferative diabetic retinopathy, T1DM – Type 1 Diabetes Mellitus, T2DM – Type 2 Diabetes Mellitus.
